# Reproducibility of peak moment for isometric and isokinetic knee extension exercise

**DOI:** 10.1186/s13102-023-00788-z

**Published:** 2023-12-16

**Authors:** Manfred Zöger, Alfred Nimmerichter, Arnold Baca, Klaus Wirth

**Affiliations:** 1 Training and Sports Sciences, University of Applied Sciences Wiener Neustadt, Johannes Gutenberg-Straße 3, 2700 Wiener Neustadt, Austria; 2 Centre for Sport Science and University Sports, University of Vienna, Auf der Schmelz 6, 1150 Wien, Austria; 3 Doctoral School of Pharmaceutical, Nutritional and Sport Sciences, University of Vienna, Josef-Holaubek-Platz 2, 1090 Wien, Austria

**Keywords:** Reproducibility, Isomed 2000, Dynamometer, Isometric, Isokinetic

## Abstract

**Background:**

Accurate measurements of muscular performance are important for diagnostics, for example during rehabilitation after traumatic injuries but also in competitive sports. For these purposes, dynamometric devices are widely used and considered the gold standard for muscle strength testing. However, few previous studies have tested the reproducibility of peak moment (PM) at velocities close to the maximum device capability, and in general, reproducibility results cannot be transferred to other devices or test protocols. The purpose of this study was to evaluate the reproducibility of PM for different isometric and isokinetic knee extension exercises using the IsoMed 2000.

**Methods:**

Thirty subjects volunteered in three repeated test sessions, including isometric knee extension (100° and 140° knee angle) and isokinetic knee extension (30°/s and 400°/s). Statistical analysis for comparison of sessions two and three included paired sample t-test, calculation of intraclass correlation coefficient (ICC) and standard error of measurement (SEM). Additionally, Bland Altman statistics and corresponding plots were created.

**Results:**

A significant difference between sessions in PM was found for isometric knee extension in one leg (140° left). Reproducibility was high for all conditions with ICC ranging from 0.964 to 0.988 and SEM in the range of 7.6 to 10.5 Nm. Bland Altman statistics revealed a bias between − 7.3 and 0.7 Nm.

**Conclusions:**

Reproducibility of PM using the IsoMed 2000 was good after an initial familiarization trial with high values of relative reproducibility. Absolute reproducibility can be interpreted as appropriate for most common practical applications.

## Introduction

Accurate measurements of muscular performance are important for diagnostics and for detecting changes in different settings. For these purposes, isokinetic dynamometry (introduced in the 1960s) is still widely used in numerous conditions and is considered the gold standard in muscle strength testing [1, 2]. Common use cases are, for example, during the rehabilitation process after traumatic injuries or to simulate different movement velocities of activities to improve the training effect but also for performance diagnostics in competitive sports [3]. In addition, the topic of interlimb strength asymmetries is of great interest, with a special focus on the prevention of injuries [4–10]. All of these issues are commonly investigated using dynamometric devices.

In the past, several studies that examined the reproducibility of dynamometric findings focused on the knee joint [11–22]. However, it is very difficult to generalize from one reproducibility study to another due to different devices, test-protocols and subject-groups. For example, Sole, Hamrén [23] examined the reproducibility for concentric and eccentric knee extension and flexion at 60°/s using a KinCom dynamometer. Dirnberger, Kösters [24] tested the reproducibility using another device and added a velocity of 120°/s to their protocol. Van Tittelboom, Alemdaroglu-Gürbüz [25] recently tested the reliability in children for isokinetic knee (and hip) flexion and extension at 60 and 90°/s. Dirnberger, Wiesinger [26] and Maffiuletti, Bizzini [27] are some of the few that tested knee extension in an isometric condition, with knee angles of 90° and 120°, respectively. In general, most of these studies indicate good reproducibility in measuring maximum muscular knee performance. The intraclass correlation coefficient (ICC) was > 0.9 in all these studies except Van Tittelboom, Alemdaroglu-Gürbüz [25], who found ICCs in the range of 0.59–0.87. This study also revealed the highest standard error of measurement (SEM) ranging from 15.7–22.8%, while the other studies found SEM < 10%.

For the current study, the IsoMed 2000 dynamometer was used. This dynamometer is capable of testing velocities up to 560°/s. To the best of the authors’ knowledge, there is currently only one study examining the reproducibility using this device at slow velocity [28] and no studies that used a velocity somewhere close to the maximum capability of the device. In addition, most previous studies that examined isometric contractions did this at only one joint angle. That said, there is currently a knowledge gap regarding the reproducibility of peak moment for very slow and very fast velocities and for different isometric joint angles when using this device.

The aim of this study was therefore to determine the reproducibility of PM for maximum isometric and isokinetic knee extension at two different joint angles (100° and 140°) and at very slow (30°/s) and very fast (400°/s) velocities using the IsoMed 2000 dynamometer.

## Methods

### Subjects

Thirty subjects (25 male, 5 female; mean (SD): stature 179.4 (8.4) cm; body mass 76.0 (9.9) kg; age 30.6 (8.2) years) with no history of orthopaedic lower extremity pathology volunteered to participate in this study. Before entering the study, all subjects were physically active on a recreational level but had no previous experience in isokinetic exercise. For consistent testing conditions, subjects were instructed not to engage in vigorous physical activity for 48 h, ingestion of caffeine for 12 h, and consumption of food for 3 h prior to each test. Before the first visit to the laboratory, subjects were informed about the benefits and risks of participating in the study. Written informed consent was provided by all subjects, and they were advised that withdrawal from the study is possible at any time. The study conformed to the Declaration of Helsinki [29] and was approved by the local research ethics board at the University of Applied Sciences Wiener Neustadt on the 5th of April 2021 (approval nr RB20210405013).

### Instruments

All tests were conducted using an IsoMed 2000-dynamometer (D. & R. Ferstl Gmbh, Hemau, Germany) in combination with the manufacturer’s unilateral knee attachment. The device was calibrated before each session according to the manufacturer’s instructions. Data recording was performed at a sampling rate of 200 Hz using the manufacturer’s computer software IsoMed analyze SP3-i51.

### Procedures

Subjects were tested in three identical sessions. As several studies recommend a familiarization trial [24, 26, 30–33], the first session was set to accommodate the participants to the device and the measurement procedure and was not included in further analysis. Tests were typically conducted 72 h apart, with a minimum of 48 h between tests, to ensure sufficient recovery between trials. Subjects were tested at the same time of day (± 1.5 h), and all tests were conducted by the same examiner to minimize possible influences from diurnal variations and inter-tester variability.

Before each session, subjects completed a standardized 10-min general warm-up. Immediately after the general warm-up, subjects were placed on the adjustable dynamometer chair with the backrest at 85° (0° = fully extended) and in a way that the popliteal fossa of the tested leg ended up with the frontal edge of the seat. The knee’s rotational axis was aligned with the dynamometer’s mechanical axis using a laser pointer, with the lateral femoral epicondyle representing a bony reference point. Adjustable straps and pads were used to achieve additional stabilization and minimize errant body movements of the subjects. Fixation included the shoulders, hip, and femur with the objective of isolating the movement of the knee joint. In addition, subjects were instructed to grip the side handles of the device with their hands. At a position of 90° knee flexion, the dynamometer lever arm and the corresponding distal shin pad were attached approximately 2.5 cm superior to the lateral malleolus using a strap. The range of motion for the knee joint was set to 90–170° (180° = fully extended). After proper placement of the subjects, individual settings were recorded by the integrated software to guarantee identical positioning in every session.

Each session consisted of isometric and isokinetic knee extensions that were measured in two conditions each and for both legs. The starting leg was randomly assigned; Starting with the right vs. starting with the left leg was evenly distributed throughout the subject group. For isometric measurements, the knee joint was fixed at knee angles of 100° (Iso100) and 140° (Iso140), and isokinetic measurements were conducted at angular velocities of 30°/s and 400°/s. The slower velocity was tested prior to the faster velocity as recommended elsewhere [34]. Initial position was achieved passively and all isokinetic measurements were completed as discrete movements in a single direction [35]. The order of tests was Iso100, Iso140, 30°/s, 400°/s, and remained the same throughout all sessions.

Prior to each condition, subjects performed a submaximal specific warm-up on the device to become accustomed to the requirements of each test. This specific warm-up consisted of 10 repetitions at an intensity corresponding to approximately 50% of maximum voluntary contraction followed by 3 repetitions at an intensity corresponding to approximately 80% of maximum voluntary contraction. After this specific warm-up, subjects received a 3-min break where the procedures for the following condition were explained via standardized instructions.

For every test condition, participants completed a minimum of three repetitions. However, additional repetitions were applied as long as PM continued to improve. All of the participants reached their PM within a maximum of five repetitions. Before each repetition, subjects received 3-min of passive rest to ensure sufficient recovery. To ensure maximum effort, visual feedback was provided on a screen in front of the participant and additional strong verbal encouragement was provided by the examiner [36, 37]. After each testing-condition, the dynamometer’s position was adapted for the other leg or the following condition.

### Statistical analysis

The repetition with the highest PM for each condition was selected [38, 39] from each session and used for analysis. Descriptive data are presented as mean (SD). The assumption of normality was verified using Shapiro-Wilk test.

Differences between PM were assessed with paired sample t-tests. Relative reproducibility was assessed with the two-way random effect ICC [40, 41] and calculated and interpreted according to Vincent [42]. Those recommendations consider an ICC over 0.9 as high, between 0.8 and 0.9 as moderate, and below 0.8 as low. For evaluation of absolute reproducibility, the SEM was calculated using the formula $SEM= SD\times \sqrt{\ 1- ICC}$ [43, 44]. In addition, the SEM% was calculated, defined as SEM/(mean of measurements from sessions) * 100. To identify the level of agreement between sessions, Bland-Altman statistics ±95% limits of agreement (LoA) were calculated and corresponding plots were created for visual presentation of individual results [45].

Statistical analyses were performed using the software package IBM SPSS Statistics for Windows, V.28.0 (IBM Corp., Armonk, N.Y., USA). Figures were created using GraphPad Prism V.9.3 for Windows (GraphPad Software, San Diego, CA, USA). The level of significance was set at *p* < 0.05.

## Results

PM was significantly higher (+ 7.2 Nm) during the third session for the Iso140 condition in the left leg (t(29) = − 2.78, *p* = 0.01). No other significant differences in peak moment between sessions were observed for any condition (Table 1).

**Table 1:** Mean (SD) for peak moment measurements as well as *p*-values for comparison of sessions

	Session 2 (Nm)	Session 3 (Nm)	*p*-value
L Iso100	263.4 (54.9)	265.5 (56.1)	0.578
R Iso100	273.5 (64.4)	272.8 (69.7)	0.817
L Iso140 *	227.5 (48.1)	234.7 (52.2)	0.010
R Iso140	234.4 (48.9)	237.0 (52.9)	0.379
L 30°/s	229.6 (49.4)	234.9 (53.3)	0.085
R 30°/s	237.9 (54.2)	245.2 (57.3)	0.053
L 400°/s	98.4 (29.9)	99.9 (30.2)	0.330
R 400°/s	98.5 (34.0)	100.4 (33.0)	0.151

L – ﻿left leg, R – ﻿right leg, Iso100 – ﻿isometric 100° knee angle, Iso140 –﻿ isometric 140° knee angle

*significant difference between session 2 and session 3 at *p* < 0.05

The ICC indicated high values of relative reproducibility, ranging from 0.964 to 0.988 (95% CI 0.921 to 0.994). Absolute reproducibility expressed as SEM and SEM% was 7.6 to 10.5 Nm and 2.8 to 7.7%, respectively (Table 2).

**Table 2:** Relative and absolute reproducibility statistics for comparison of peak moment from sessions 2 and 3

	ICC (2,1)	95% CI	SEM (Nm)	SEM (% of mean)
L Iso100	0.967	0.931–0.984	10.0	3.8
R Iso100	0.987	0.972–0.994	7.6	2.8
L Iso140	0.975	0.935–0.989	7.9	3.4
R Iso140	0.975	0.948–0.988	8.0	3.4
L 30°/s	0.972	0.941–0.987	8.5	3.7
R 30°/s	0.964	0.921–0.983	10.5	4.4
L 400°/s	0.982	0.963–0.992	7.6	7.7
R 400°/s	0.988	0.975–0.994	7.6	7.6

L – left leg, R – right leg, Iso100 – isometric 100° knee angle, Iso140 – isometric 140° knee angle, ICC – intraclass correlation coefficient, CI – confidence interval, SEM – standard error of measurement

**Fig. 1:**
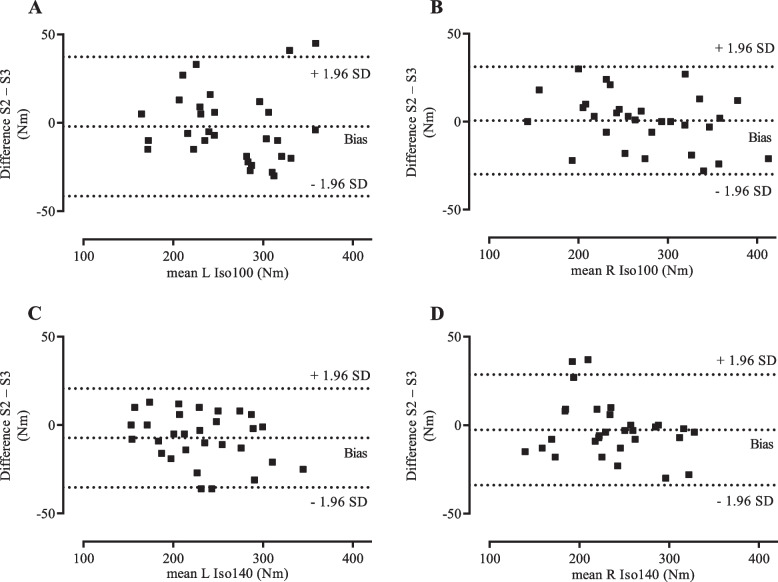
Bland-Altman plots – differences between session two and session three plotted against the means. Differences between session two and session three plotted against the means of session two and session three for **A** left leg isometric extension at 100° knee angle; **B** right leg isometric extension at 100° knee angle; **C** left leg isometric extension at 140° knee angle; and **D** right leg isometric extension at 140° knee angle

**Fig. 2:**
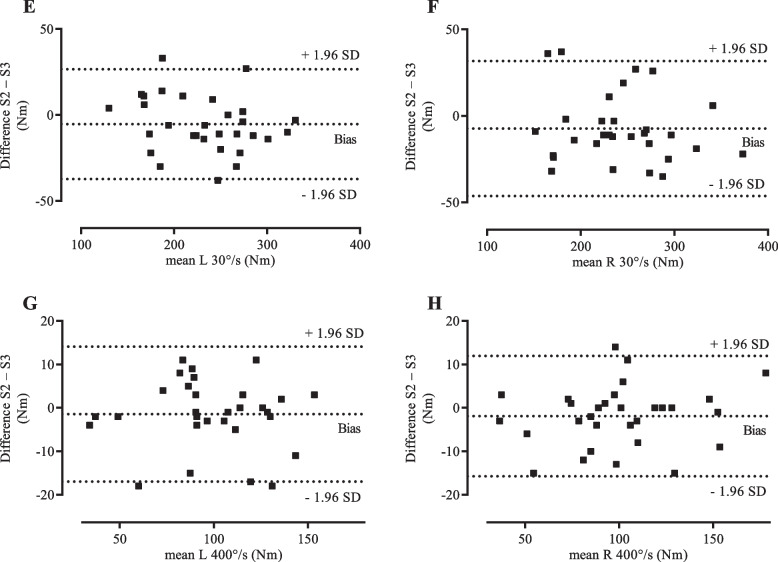
Bland-Altman plots – differences between session two and session three plotted against the means. Differences between session two and session three plotted against the means of session two and session three for **E** left leg isokinetic extension at 30°/s; **F** right leg isokinetic extension at 30°/s; **G** left leg isokinetic extension at 400°/s; and **H** right leg isokinetic extension at 400°/s

Bland-Altman plots for isometric and isokinetic knee extension illustrated a random relationship between the individual differences and the averages of sessions (Figs. 1 and 2). The bias, which represents the average difference between sessions (Table 3), ranged from − 7.3 to 0.7 Nm (95% LoA from − 46.3 to 37.3 Nm), with a negative value indicating that session three had a higher value than session two.

**Table 3:** Bias and 95% LoA for comparison of sessions 2 and 3 received from Bland-Altman calculations

	Bias (Nm)	95% LoA (Nm)
L Iso100	−2.1	−41.5–37.3
R Iso100	0.7	−29.9–31.3
L Iso140	−7.2	−35.2–20.7
R Iso140	−2.6	−33.9–28.7
L 30°/s	−5.3	−37.2–26.6
R 30°/s	−7.3	−46.3–31.7
L 400°/s	−1.4	−17.0–14.1
R 400°/s	−1.9	−15.7–11.9

L – left leg, R – right leg, Iso100 – isometric 100° knee angle, Iso140 – isometric 140° knee angle, LoA – limits of agreement

## Discussion and implications

The purpose of this study was to evaluate the reproducibility of peak moment during maximum isometric and isokinetic knee extension using the IsoMed 2000 dynamometer. In general, the results suggest a high reliability of peak moment within the conditions of the current study. However, reliability results for a specific test have to be interpreted individually, according to the analytical goals of the test [43].

A possible limitation of this study was the small female sample size. The aim of this study was to gain insights into the reproducibility of PM within human adults. Therefore, we recruited a mixed gender subject group and pooled the dataset. Unfortunately, only five female volunteered for participation in this study. This could possible cause problems regarding homogeneity of the dataset. However, deleting the female dataset did not have a dramatic effect on homogeneity and therefore we did not delete their data from this study.

An additional possible limitation is the fact that we did not calculate an optimal sample size for our study. Instead we aimed for the maximum number of subjects that were available within the scheduled time-period of this study. All subjects of our study were physically active on a recreational level but did not have any previous experience in isokinetic exercise. That said subjects with previous experience in isokinetic exercise or on a higher performance level could possibly obtain other results.

An important note is that we used the first session as a familiarization session, as recommended by several authors [24, 26, 30–33]. The main goal of our study was to determine the reproducibility of peak moment (after initial familiarization). Therefore, we analysed and compared the values for peak moment from session two and session three and did not report the values from the initial familiarization session.

We found no significant difference between sessions two and three, except for Iso140 in the left leg. This finding is similar to Impellizzeri, Bizzini [46], who also found a systematic time effect in one condition of the left leg. However, Impellizzeri, Bizzini [46] detected a significant difference for concentric extension at 180°/s. A possible reason for this identified difference in the left leg could be leg dominance, though this was not analysed in our study. Therefore, the exact reason for the significant difference in only one condition of the left leg remains unclear.

Generally, our results show very good reproducibility of peak moment using the IsoMed 2000 after an initial familiarization. Regarding relative reproducibility we found ICC values > 0.964 with 95% CI of 0.921 and higher. Between all the conditions of our protocol, we detected only small differences in ICC. Therefore the relative reproducibility can be estimated as high for all conditions in this study, according to the recommendations of Vincent [42].

According to Caruso, Brown [47], the excellent levels of reproducibility under test-retest conditions seem to be unique for the knee joint.

When comparing our results to previous studies, slightly higher results were observed. The ICC values in our study are higher than those obtained in a similar study by Kues, Rothstein [48], who tested isometric knee angles of 120° and 140°. Our results also show higher ICC than in the study of Alt, Knicker [28]. These authors found values < 0.9 for the comparison of T2-T3 in isokinetic knee extension. However, in their study subjects were tested in a supine position that was different from the seated position in our study. Our results for ICC are similar to those of Dirnberger, Kösters [24], who found ICC values in the range of 0.976–0.984 for concentric isokinetic knee extension at velocities of 60°/s and 120°/s, respectively.

In our study, we found better relative reproducibility (ICC) for the faster velocity which is contrary to previous research results. For example, Brown, Whitehurst [49] tested a broad range of different isokinetic velocities from 60 to 450°/s. Regarding knee extension, they found a trend for better reproducibility at slower velocities. The same was true in the study of Fagher, Fritzson [50] who also found better relative reproducibility for slower velocity. It should be noted, however, that the participants in their study were children aged 8 to 10 years, which could impair direct comparisons with adults.

Regarding absolute reproducibility, almost all obtained values in our study are below 10 Nm, with only Iso100 for the left leg (10.0 Nm) and 30°/s for the right leg (10.5 Nm) being just slightly higher (Table 2). When comparing the SEM (Nm) for slow and fast isokinetic velocities, it was lower for 400°/s compared to 30°/s. However, calculating the SEM% revealed values between 2.8 and 4.4% for all isometric conditions and for 30°/s. At 400°/s the SEM% was considerably higher (7.6 and 7.7% for the right and left leg, respectively). This is almost twice that of most of the other conditions and would indicate inferior reproducibility for the faster velocity of 400°/s. These results for absolute reproducibility are similar to those found by Dirnberger, Wiesinger [26]., although the authors in this study tested only one leg. In contrast to these results that found better reproducibility for slower velocity, other authors [24, 26, 27, 48, 51] have found better reproducibility for faster velocities. On the other hand, Impellizzeri, Bizzini [46] found no clear tendency regarding velocity. The authors reported the lowest SEM% for 180°/s, followed by 60°/s. For 120°/s, the SEM% was the highest of the three conditions in this study. However, the difference between the three tested velocities of their protocol was small, and the range between the slowest and the fastest velocity regarding SEM% was just 0.8%.

Therefore, based on current available studies and the results of this study, there is inconsistency regarding a comparison between reproducibility results of different velocities for isokinetic knee extension. That points out that generalizing reproducibility results is difficult and that different velocities lead to partly divergent reproducibility.

## Conclusion

This study investigated the reproducibility of peak moment obtained from different isometric and isokinetic knee extension exercises using the IsoMed 2000 dynamometer. The findings suggest that after an initial familiarization trial, peak moment can be measured at a level that is considered excellent for most common applications. Practitioners should aim for a familiarization trial in each angle and velocity when doing isometric or isokinetic exercise to obtain reliable results.

## Data Availability

The datasets used and analysed during the current study are available from the corresponding author on reasonable request.

## Abbreviations

ICC: intraclass correlation coefficient
Iso100: isometric, 100° knee angle
Iso140: isometric, 140° knee angle
LoA: limits of agreement
PM: peak moment
SEM: standard error of measurement
